# Editorial: Perspectives in veterinary and zoonotic infection: 2025

**DOI:** 10.3389/fcimb.2026.1940121

**Published:** 2026-08-20

**Authors:** Carissa Embury-Hyatt, Mohamed A. Abouelkhair

**Affiliations:** 1 National Centre for Foreign Animal Disease, Canadian Food Inspection Agency (CFIA), Winnipeg, MB, Canada; 2 Department of Veterinary Biomedical Sciences, Rowan University, Glassboro, NJ, United States

**Keywords:** antimicrobial resistance, bacterial virulence, one health, vaccine, viral persistence, zoonotic pathogens

The convergence of animal and human health represents one of the defining challenges of infectious disease epidemiology and one health. Zoonotic pathogens account for the substantial proportion of emerging human infections, with livestock, companion animals, and wildlife serving overlapping roles as reservoirs, amplifying hosts, and epidemiological sentinels. In this context, the capacity to molecularly and phenotypically characterize pathogens and develop effective countermeasures in animal populations has never been more critical. This Research Topic brings together ten contributions published in *Frontiers in Cellular and Infection Microbiology Veterinary and Zoonotic Infection* that collectively advance our understanding of bacterial and viral pathogens at the veterinary–human interface, with particular emphasis on virulence mechanisms, antimicrobial resistance, host-pathogen dynamics, and translational control strategies.

A prominent theme across several articles is the molecular basis of bacterial pathogen success in animal hosts. Wang et al. employed RNA sequencing of avian pathogenic *Escherichia coli* (APEC) isolated from the yolk of experimentally infected chicken embryos, alongside *in vitro* grown controls, and demonstrated that APEC deploys distinct, host-context-dependent transcriptional programs during embryonic infection. In embryos that succumbed, APEC exhibited a transcriptionally inferred hypermetabolic state, including enhanced broad nutrient acquisition, anaerobic respiration, and purine and arginine biosynthesis. In surviving embryos, APEC instead displayed profiles indicative of host-imposed iron restriction, envelope stress. These findings support a model in which infection outcome is governed by the bacterium’s capacity to compete for host-derived nutrients rather than by discrete virulence loci, positioning bacterial energy metabolism and iron acquisition pathways as rational targets for future intervention strategies.

The Research Topic also documents the capacity of bacterial species previously considered of limited clinical relevance to cause severe disease across the animal–human divide. Shi et al. report the first fatal human bloodstream infection attributable to *Macrococcus caseolyticus* subsp. *caseolyticus* in China, supported by whole-genome sequencing that revealed an unexpectedly rich complement of virulence determinants and antimicrobial resistance genes, corroborated by phenotypic susceptibility profiling that confirmed clinically relevant resistance. This case challenges assumptions about the pathogenic potential of environmental staphylococcal relatives and has implications for surveillance in clinical and veterinary microbiology alike. Antimicrobial resistance in livestock-associated pathogens with direct zoonotic relevance is further addressed by Elsayed et al., whose genomic characterization of *Mycobacterium bovis* strains from cattle in Egypt’s Delta region reveals high genetic diversity and resistance profiles, complicating control efforts. Bovine tuberculosis at the livestock–wildlife–human interface remains a persistent One Health challenge, and genotypic data of this kind are essential for designing effective eradication and surveillance programs in regions where exposure risk to human populations remains high.

Ma et al. identified Type A and D *Clostridium perfringens* strains with distinctive genomic evolution characteristics as the cause of fatal disease in juvenile Bactrian camels. Bactrian camels occupy an important economic and ecological role in arid and semi-arid areas and characterizing the clostridial disease agents affecting this species enriches the comparative microbiology and provide a foundation for improved diagnosis, prevention, and vaccine development. The complexity of mixed infections at the human–animal interface is addressed by Zhang et al., who demonstrate that co-infection with the zoonotic pathogen *Chlamydia psittaci* and the commensal opportunist *Enterococcus faecalis results in* more severe respiratory disease in patients than either organism alone, a synergy they validate using a mouse co-infection model. This study underscores that zoonotic events frequently unfold against a background of existing microbial communities and that syndromic molecular diagnostic approaches may be necessary to capture the full infectious picture. Complementing studies on bacterial infections, Chen et al. demonstrate that the indole-3-carboxamide attenuates LPS-induced endometritis in mice by suppressing ferroptosis and inflammatory signaling via modulation of the aryl hydrocarbon receptor.

Viral contributions to this Research Topic span fundamental biology, methodological rigor, and field-deployable prevention. Li and Pickering provide a timely review of viral RNA persistence and long-term clinical consequences following acute infection with Risk Group 4 zoonotic pathogens, including filoviruses and henipaviruses. As survivor cohorts from major outbreak responses have grown, evidence has accumulated that RNA persistence in immune-privileged compartments drives long-term sequelae, recrudescence, and ongoing transmission-risk challenges, which this review synthesizes with clarity for researchers and public health practitioners working at the high-consequence end of the zoonotic spectrum. A methodological complement to this virology work is provided by Kim and Pickering, whose mini-review on cell line bias in virus research examines how the choice of *in vitro* model shapes viral propagation efficiency and phenotypic interpretation. Their analysis carries practical implications for experimental design across veterinary and human virology, particularly where *in vitro* data are intended to inform regulatory decisions or clinical inference.

Swine influenza, a perennial pandemic candidate, is examined by Arranz-Herrero et al. from the perspective of the host pulmonary microbiome. Their finding that swine influenza virus infection substantially remodels the lower respiratory microbial community advances our understanding of how primary viral infection creates a permissive landscape for secondary bacterial disease, with clear parallels to influenza-associated bacterial pneumonia in human medicine and direct implications for swine herd management. Currently there is great interest in unravelling the role of microbiota in viral infections, given that it could be a potential therapeutic target or an important prognostic marker. Finally, Mhamadi et al. report that a DNA vaccine candidate confers protection against Rift Valley Fever virus in sheep under natural field conditions in West Africa, a significant translational advance for a pathogen causing a self-limiting febrile illness in humans (though severe and fatal in neonates/rare cases) and catastrophic reproductive losses in ruminants. Field efficacy data of this kind are essential for regulatory pathways and national vaccination programs in endemic and at-risk regions.

Taken together, the contributions to this Research Topic demonstrate that progress in veterinary and zoonotic infection science requires an integrated approach. Molecular epidemiology and host–pathogen interaction studies can reveal both diversity and the potential for emergence; mechanistic work on virulence and host immunity can illuminate intervention targets; and improved diagnostics and field-deployable countermeasures can translate scientific findings into practical disease control. Crucially, the articles assembled here collectively affirm that animal and human health are inseparable. Future work will benefit from expanded genomic surveillance and more systematic investigation of co-infection dynamics and microbiome pathogen interactions as determinants of zoonotic risk and disease severity.

